# Marine Bioactive Compounds from Functional Seafoods: Pharmacological Mechanisms and Health Applications

**DOI:** 10.3390/md24030116

**Published:** 2026-03-20

**Authors:** Sena Davran Bulut, Naciye Yaktubay Döndaş, Senanur Koçhan, Beyza Nur Arslan, Mehmet Ali Tamer, Mirsade Osmani, Safa Baraketi, Khaoula Khwaldia, Ziye Zhang, Hacı Ali Döndaş, Tuba Esatbeyoglu, Panagiota Katikou, Fatih Ozogul

**Affiliations:** 1 Department of Biotechnology, Institute of Natural and Applied Sciences, Çukurova University, Balcalı, 01330 Adana, Türkiye; sena.davran7@gmail.com (S.D.B.); yakdas25@cu.edu.tr (N.Y.D.); senanurtas99@gmail.com (S.K.); mali.tamer88@gmail.com (M.A.T.); adondas@cu.edu.tr (H.A.D.); 2 Department of Biotechnology, Faculty of Science, Bartin University, 74100 Bartin, Türkiye; 3 Department of Medical Pharmacology, Faculty of Medicine, Çukurova University, Balcalı, 01330 Adana, Türkiye; bnarslan@cu.edu.tr; 4 Department of Translational Medicine, Institute of Health Sciences, Çukurova University, Balcalı, 01330 Adana, Türkiye; 5 Department of Basic Pharmaceutical Sciences, Faculty of Pharmacy, Çukurova University, Balcalı, 01330 Adana, Türkiye; 6 Faculty of Food Technology, University of Mitrovica “Isa Boletini”, Str. Ukshin Kovacica, 40000 Mitrovica, Kosovo; mirsade.osmani@umib.net; 7 Laboratory of Natural Substances (LSN), National Institute of Research and Physicochemical Analysis (INRAP), BiotechPole Sidi Thabet, Sidi Thabet 2020, Tunisia; safabaraketi@outlook.com (S.B.); khaoula_khwaldia@yahoo.fr (K.K.); 8 State Key Laboratory of Marine Food Processing & Safety Control, College of Food Science and Engineering, Ocean University of China, Qingdao 266404, China; zhangziye@ouc.edu.cn; 9 Department of Advanced Materials and Nanotechnology, Institute of Natural and Applied Sciences, Çukurova University, Balcalı, 01330 Adana, Türkiye; 10 Department of Molecular Food Chemistry and Food Development, Institute of Food and One Health, Gottfried Wilhelm Leibniz University Hannover, Am Kleinen Felde 30, 30167 Hannover, Germany; esatbeyoglu@foh.uni-hannover.de; 11 Veterinary Research Institute of Thessaloniki, Hellenic Agricultural Organization—DIMITRA, Ktima Thermis, 57001 Thessaloniki, Greece; 12 Department of Seafood Processing Technology, Faculty of Fisheries, Çukurova University, Balcalı, 01330 Adana, Türkiye

**Keywords:** functional seafoods, marine bioactive compounds, nutraceuticals, omega-3 fatty acids, pharmacological mechanisms, sustainable aquaculture, chronic disease prevention

## Abstract

Functional seafoods derived from marine organisms, including fish, shellfish and algae, are gaining increasing attention due to their high content of bioactive compounds, such as omega-3 fatty acids, peptides, polysaccharides and antioxidants, which provide health benefits beyond basic nutrition. These marine-derived compounds exhibit a wide range of biological activities and have been investigated for their potential roles in the prevention and management of chronic diseases, including cardiovascular, neurodegenerative, cancer and gastrointestinal disorders. Their effects are largely mediated through anti-inflammatory, antioxidant and immunomodulatory mechanisms. Advances in biotechnology, including genetic engineering and improved extraction of bioactive compounds, have enhanced the nutritional quality and pharmacological relevance of functional seafoods. At the same time, sustainable aquaculture practices are being developed to reduce environmental impacts. Nevertheless, challenges such as regulatory inconsistencies, scalability issues and limited understanding of bioavailability and long-term effects still persist. These constraints should be considered when interpreting mechanistic and efficacy findings presented across different study designs and exposure conditions. Future perspectives highlight innovations in precision aquaculture, waste valorisation and traceability as key strategies to improve sustainability and strengthen consumer trust. This review summarizes current knowledge on functional seafoods, with emphasis on pharmacological mechanisms, clinical applications and the need for interdisciplinary research to optimize their health benefits and commercial potential.

## 1. Introduction

As health and wellness become central priorities in research, policy and product development, global interest in functional foods has significantly increased. At the cutting edge of this nutritional awareness stand functional seafoods, distinguished not only for their sensory qualities but also for their potential health benefits due to their rich nutritional profile and unique bioactive compounds [1].

Functional seafoods include marine-derived foods that offer health benefits beyond basic nutrition. This category includes diverse products and matrices such as bioactive peptides, fish oils, microalgae, and fish proteins, which occur in various marine sources such as fish, shellfish and algae. Additionally, these foods contain bioactive compounds that are associated with potentially beneficial physiological effects [2,3].

Due to their richness in omega-3 fatty acids, fatty fish such as salmon, mackerel and sardines have been extensively studied for their role in reducing inflammation and decreasing the risk of chronic diseases [4]. Microalgae and cyanobacteria, such as *Chlorella* spp. and *Spirulina* spp., are recognized for their high protein content and antioxidant properties, which may contribute to improved immune function and overall health [5]. Beyond these well-known examples, functional seafoods include a wide variety of other marine organisms. For instance, sea cucumbers have been studied for anti-inflammatory and anti-tumor properties [6], while seaweeds such as kelp and nori are rich in vitamins, minerals and polysaccharides that may promote gut health and metabolic function [7,8]. The category of functional seafoods also includes products derived from marine bioprocessing, such as fish protein hydrolysates and marine collagen, which contain bioactive peptides and have been associated with potential health benefits [1].

The emerging field of biotechnology has provided new insights and innovations, further highlighting the importance of functional seafoods in supporting human health. This increasing interest reflects not only evolving consumer preferences but also a growing recognition of the role of diet, including functional seafoods, in preventing and managing disease. Researchers have highlighted potential roles in reducing the risk of chronic diseases such as cardiovascular diseases, inflammation and certain types of cancer [9]. Moreover, functional seafoods are being studied for their role in supporting mental health, enhancing immune function, and contributing to overall well-being [10]. For example, omega-3 fatty acids have been associated with reduced rates of depression and anxiety, suggesting that regular consumption of fatty fish may influence mental health outcomes [3].

Functional seafoods may also contribute to addressing nutritional deficiencies in populations with limited access to diverse food sources. In many developing countries, seafood provides an essential source of protein, vitamins and minerals, contributing to nutritional security and public health [11]. Therefore, integrating functional seafoods into dietary guidelines and public health strategies may help combat malnutrition and improve health outcomes.

Recent advancements in biotechnology have further amplified the potential of functional seafoods. Techniques such as genetic modification, fermentation and extraction of bioactive compounds can enhance nutritional and therapeutic attributes [3]. For instance, genetic modification can increase levels of beneficial compounds in seafood [12], while fermentation processes may improve bioavailability, making nutrients easier for the body to absorb. The extraction of bioactive peptides from fish by-products has also shown potential for creating value-added products with health benefits [13]. These innovations may additionally support sustainability by reducing waste and enabling new functional foods with enhanced nutritional profiles.

Despite progress, several challenges remain, particularly regarding sustainable sourcing, standardization of bioactive components and ensuring consumer safety and acceptance [11]. Sustainable sourcing of functional seafood is a critical issue that must be addressed to ensure long-term availability. Overfishing and environmental degradation pose significant threats to marine ecosystems, highlighting the need for sustainable aquaculture practices and responsible harvesting methods [14]. Furthermore, standardization of bioactive components is crucial for ensuring consistency and efficacy of functional seafood products. Regulatory frameworks are needed to support quality control and to safeguard consumers from potential risks [11].

Another challenge lies in existing gaps in our understanding of functional seafoods. Despite the growing body of literature, important uncertainties remain. While numerous studies have underscored potential health benefits, more clinical trials are still needed to provide clear evidence and to define the conditions under which benefits are most likely to occur. For example, the anti-inflammatory properties of omega-3 fatty acids are well known; however, the optimal dosage and long-term effects on specific health conditions remain insufficiently defined [3]. In addition, the bioavailability and metabolism of bioactive compounds in functional seafoods may vary considerably among individuals, which complicates assessment of their health effects [15].

Additionally, the mechanisms of action of many bioactive compounds remain incompletely understood. Understanding molecular pathways involved in functional seafood-related effects is crucial for developing targeted interventions. For example, cardioprotective effects of omega-3 fatty acids have been linked to reduced inflammation and lowered blood pressure, but the precise molecular mechanisms responsible for these effects are still being investigated [3].

Moreover, the interaction between bioactive compounds in functional seafoods and other dietary components remains an important area for further study. Individual compounds such as omega-3 fatty acids and peptides have been studied thoroughly; however, their synergistic effects when consumed within whole diets are not fully understood. Better characterization of these interactions is essential for developing dietary recommendations that maximize potential benefits [16]. Another important factor is the effect of cooking and processing methods on the stability and activity of bioactive compounds in functional seafoods. Conventional methods such as boiling, grilling and frying can alter the nutritional composition of seafood and may affect the stability and bioactivity of beneficial compounds [17]. Therefore, optimized processing techniques are needed to preserve the integrity of bioactive compounds while ensuring food safety and consumer acceptance.

The present review aims to provide a thorough and structured analysis of the current state of knowledge, highlighting recent findings and opportunities for future research. In contrast to reviews that focus primarily on nutritional benefits or individual compound classes, this manuscript adopts an integrative pharmacological framework with clear translational orientation. The nutritional and bioactive components of functional seafoods are discussed, along with their potential health benefits and recent advancements in biotechnological methods used to enhance their properties. Furthermore, this review examines pharmacological mechanisms at the molecular level, includes clinically approved marine-derived therapeutic agents and addresses the relevant mechanistic pathways and translational implications. By synthesizing recent findings, integrating functional seafoods with marine-derived pharmaceuticals within a translational perspective and identifying research gaps, this review seeks to support future research and innovation in the field of functional seafoods.

## 2. Literature Identification and Evidence Evaluation Framework

### 2.1. Literature Identification and Selection Strategy

This review was developed as a structured narrative synthesis of the literature on functional seafoods and marine-derived bioactive compounds. Relevant publications were identified through targeted searches of PubMed, Scopus and Web of Science using combinations of appropriate keywords, such as: “functional seafood”, “marine bioactive compound”, “marine-derived drug”, “omega-3 fatty acid”, “marine polysaccharide”, “marine peptide”, “nutraceutical”, “cardiovascular disease”, “neurodegenerative disease”, “cancer”, “health effect”, “mechanism” and “clinical study”. Boolean operators (AND, OR) and truncation symbols (*) were used to combine search terms and refine retrieval where appropriate. Indicative examples of search strings include: (i) (“functional seafood” OR “marine bioactive compound*”) AND (“health effect*” OR “mechanism*” OR “clinical stud*”); (ii) (“omega-3 fatty acid*” OR “marine polysaccharide*” OR “marine peptide*”) AND (“cardiovascular disease*” OR “neurodegenerative disease*” OR cancer), and; (iii) (“marine-derived drug*” OR nutraceutical*) AND (“health effect*” OR “mechanism*” OR “clinical stud*”). The search strings were adapted for each database to account for differences in indexing systems and query syntax.

Priority was given to peer-reviewed original research articles, clinical studies and authoritative review papers addressing mechanistic insights, experimental validation, or human evidence. Inclusion criteria entailed publications in the English language, published primarily within the last two decades (approximately 2000–2025), with the majority of references originating from the most recent decade. The retrieved records were initially screened based on title and abstract to assess relevance to the thematic scope of the review. Publications clearly unrelated to marine-derived compounds or functional seafoods were excluded at this stage. The full text of potentially relevant articles was subsequently examined and studies were selected based on their contribution to mechanistic understanding, experimental validation or clinical relevance. Particular emphasis was placed on studies providing quantitative experimental data, clearly defined biological endpoints or human intervention evidence, where available. Publications that did not address marine organisms or marine-derived bioactive compounds, or that focused exclusively on unrelated nutritional interventions, were excluded during the screening process. Conference abstracts, editorials and publications lacking sufficient experimental or clinical relevance to the health effects of marine-derived bioactive compounds were also not considered further.

Reviews were used primarily to contextualize broader mechanistic frameworks, while primary research articles were preferentially cited where available. Reference lists of relevant review articles were also examined to identify additional key publications not captured through the database searches. When multiple studies addressed similar mechanisms or clinical outcomes, preference was given to studies providing quantitative data, well-defined experimental conditions or human intervention evidence. During the interpretation of the selected literature, attention was given to the study design and experimental context of the reported findings. In particular, distinctions were made between in vitro experiments, animal studies and human research when discussing biological mechanisms or health outcomes. Potential limitations, including small sample sizes, highly controlled experimental conditions and limited replication across studies, were also considered when evaluating the strength and relevance of the available evidence.

### 2.2. Evidence Classification and Interpretive Framework

**Figure 1:**
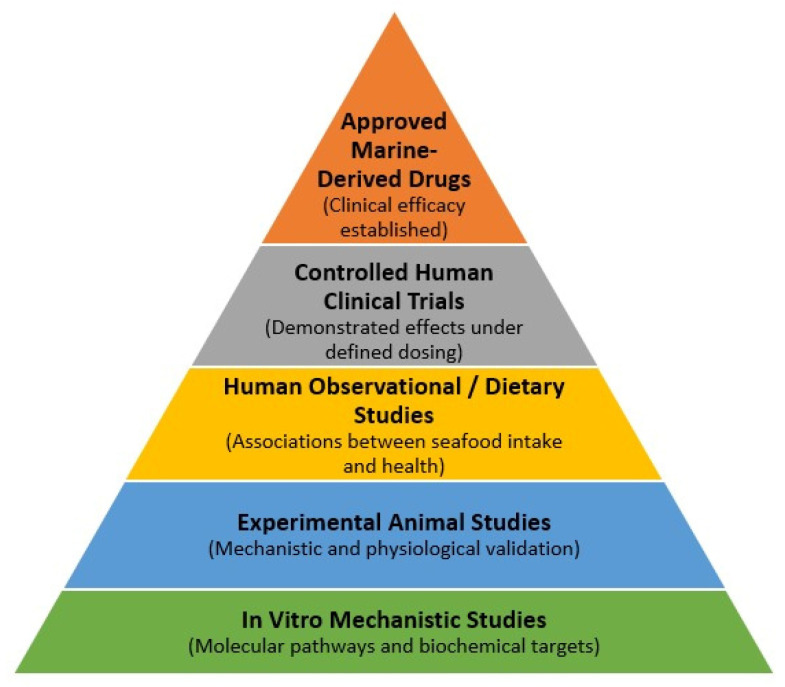
Hierarchical framework of evidence supporting health effects of marine-derived bioactive compounds.

The literature identified through the search process encompasses heterogeneous levels of evidence, ranging from mechanistic in vitro studies and experimental animal models to human observational studies, clinical trials and approved marine-derived pharmaceuticals. To improve interpretability, evidence discussed throughout this review is presented within a hierarchical framework reflecting increasing levels of biological and clinical validation (Figure 1).

This framework illustrates the progression from mechanistic evidence towards increasing levels of clinical validation and highlights the distinction between dietary exposure studies and formally developed marine-derived pharmaceuticals. Mechanistic studies provide important insights into molecular pathways and biological targets, whereas findings from human observational or intervention studies provide more direct evidence regarding potential health relevance. Nevertheless, mechanistic findings derived from in vitro or experimental models require cautious interpretation and should not be directly extrapolated to dietary exposure or nutraceutical use without supporting human evidence. In addition, marine-derived pharmaceutical agents discussed in this review represent a distinct category, reflecting compounds that have undergone formal drug-development pathways, including clinical trials and regulatory approval. These agents are therefore considered separately from dietary seafood consumption or nutraceutical supplementation.

In the sections to follow, mechanistic findings are discussed with reference to their corresponding level of evidence and, where available, their relevance to human exposure and clinical outcomes. For clarity, three distinct but related domains are considered throughout this review: (i) dietary intake of whole seafood as part of habitual nutrition, (ii) purified or standardised marine-derived nutraceutical preparations and (iii) marine-derived pharmaceutical agents approved for clinical use. These domains differ in regulatory status, dosing strategies, clinical endpoints and standards of evidence. Accordingly, mechanistic and therapeutic claims discussed in this manuscript are interpreted within the appropriate evidentiary and regulatory context.

## 3. Functional Seafoods and Marine Bioactives in Human Health

### 3.1. Functional Seafoods as Dietary Sources of Bioactive Compounds

Beyond basic nutrition, functional seafoods provide a rich source of bioactive components that may promote human health. Compounds such as omega-3 fatty acids, proteins, polysaccharides and polyphenols have been associated with a reduced risk of long-term diseases, including cardiovascular disorders, neurodegenerative conditions, metabolic disturbances and chronic inflammation [18]. Through their antioxidant and anti-inflammatory properties, these bioactive constituents may contribute to a reduction in oxidative stress and support cellular repair mechanisms, which are essential for long-term health maintenance [19,20].

Previous researchers have reported associations between regular consumption of functional seafoods and improved health outcomes, including cardiovascular health, cognitive function and reduced inflammatory status [21]. Among marine bioactives, omega-3 fatty acids, particularly eicosapentaenoic acid (EPA) and docosahexaenoic acid (DHA), are among the most extensively studied and are well known for their cardioprotective properties [22,23]. In addition, bioactive peptides derived from marine proteins have been shown to inhibit angiotensin-converting enzyme (ACE), a key regulator of blood pressure, thereby exhibiting antihypertensive effects. Marine-derived compounds have also been reported to support synaptic plasticity and protect neuronal cells from oxidative damage, suggesting a potential role in neuroprotection [24].

These bioactive components may be particularly relevant for older adults, individuals at risk of metabolic syndrome and populations seeking preventive health strategies [25]. Incorporation of seafood-derived dietary supplements into the diet facilitates the intake of essential nutrients, including omega-3 fatty acids, which are important for cardiovascular and neurological health [26]. The marine environment encompasses a wide diversity of organisms, such as fish, mollusks, crustaceans, and algae, each of which contains distinct bioactive molecules with potential health benefits [27]. The concentration and composition of these bioactive compounds are influenced by factors such as habitat conditions, species characteristics and environmental variables, contributing to the nutritional value and bioavailability of seafood-derived nutrients [28].

Functional seafoods may also play a role in weight management and obesity prevention. Bioactive compounds derived from fish, including chitosan and omega-3 fatty acids, have been reported to influence lipid metabolism and appetite regulation [29]. Chitosan, obtained from shellfish exoskeletons, has been shown to reduce dietary fat absorption in the gastrointestinal tract, supporting its use in weight control formulations [23]. Furthermore, omega-3 fatty acids have been linked with improved glucose regulation and enhanced insulin sensitivity, which are important factors in the prevention of type 2 diabetes [30]. Fish protein hydrolysates may additionally promote satiety and reduce overall caloric intake, thereby contributing to metabolic balance and weight control [31].

Seafood-derived bioactive molecules have also demonstrated antimicrobial and immunomodulatory properties. Marine polysaccharides, such as fucoidan and laminarin, have been reported to enhance immune function by promoting immune cell activity and modulating inflammatory responses [32]. These properties are of particular interest in the context of increasing antibiotic resistance, as marine bioactives may offer alternative or complementary strategies for immune support. Furthermore, certain studies suggest that marine-derived compounds contribute to the maintenance of gut microbiota balance, promoting the growth of beneficial microorganisms and supporting digestive and immune health [25]. Increased gut microbiome diversity associated with dietary omega-3 intake may further enhance host immune defense.

Ongoing research continues to explore novel bioactive compounds from deep-sea organisms and microalgae with reported anti-inflammatory, antioxidant and metabolic regulatory properties. These efforts are expected to further expand the range and applications of functional seafoods [3]. Accordingly, the global market for functional seafood-based products is projected to grow, as awareness of their potential health benefits increases [33]. Governments, academic institutions and industry stakeholders are increasingly collaborating to support research, innovation and policy development aimed at maximizing the health benefits of seafood-derived nutraceuticals, while ensuring environmental sustainability [34]. Awuchi et al. highlighted the growing interest in the application of seafood-based functional foods within medical nutrition therapy, particularly for the dietary management of chronic conditions such as inflammatory diseases and diabetes [35]. Table 1 presents a summary of commonly consumed seafoods, including their key nutrients and associated health benefits.

### 3.2. Marine Bioactive Compounds in Nutraceutical and Functional Product Formulations

Marine polysaccharides, including fucoidan, carrageenan and alginate, have been reported to support gastrointestinal health and regulate inflammatory responses, partly through prebiotic activity. Advances in food science and processing technologies have enabled the incorporation of marine-derived nutraceuticals into a variety of functional food products, including fortified seafood items, omega-3-enriched supplements and protein-based dietary formulations [45]. Raposo et al. emphasized that sustainable aquaculture practices and environmentally friendly processing methods play an important role in maintaining the long-term availability, stability and functionality of seafood-derived bioactive compounds used in functional foods [23].

Marine protein hydrolysates are increasingly utilized as nutritional supplements due to their potential to support muscle recovery, metabolism and weight management [46]. Similarly, marine-derived collagen peptides have gained importance in functional foods and cosmeceuticals, as they have been associated with improvements in skin elasticity, wound healing, joint health and connective tissue maintenance [19]. These peptides have been reported to enhance skin hydration and reduce signs of aging by stimulating endogenous collagen synthesis [47]. In addition, seafood-derived peptides exhibit antioxidant and anti-inflammatory properties, which may support skin repair and help mitigate damage caused by environmental stressors, such as ultraviolet radiation and pollution [48]. Omega-3 fatty acids have also been shown to contribute to skin barrier function, reducing transepidermal water loss and supporting overall skin health [25].

### 3.3. Sustainability and Technological Considerations Relevant to Functional Seafood Products

The sustainability of marine-based functional foods represents a growing concern due to increasing demand for seafood-derived bioactive compounds [47]. Researchers have emphasized the importance of responsible aquaculture practices, renewable resource management and sustainable extraction technologies to balance environmental protection with the production of high-quality functional foods [49]. Pattnaik et al. reported that advanced processing techniques, including enzymatic hydrolysis, microencapsulation and cold-press extraction, may improve the stability and bioefficacy of marine-derived bioactives [50]. In parallel, developments in algae cultivation and aquaponic systems are contributing to more sustainable production pathways for marine bioactive compounds [23].

## 4. Pharmacological Applications of Marine Bioactive Compounds from Functional Seafood Sources

The compounds discussed in this section represent marine-derived pharmaceutical agents and advanced translational candidates developed under formal drug-development pathways and should not be equated with dietary seafood consumption or nutraceutical supplementation. Beyond dietary consumption and nutraceutical use, many medicines currently in clinical use are derived from natural compounds and/or their derivatives, which have long been recognized as valuable sources for drug discovery and development due to their biological activity and structural diversity. In addition to terrestrial plants, animals and microorganisms, marine-derived compounds associated with functional seafood sources are gaining popularity due to their unique chemical and structural properties and their promising therapeutic potential [51]. While functional seafoods are primarily consumed as whole foods with health-promoting properties, it is important to distinguish these dietary products from isolated marine-derived bioactive molecules and clinically approved pharmaceuticals. These categories differ substantially in terms of regulatory framework, dosage standardization, intended use, and clinical validation.

Seafood-derived nutrients and bioactive compounds exhibit a wide range of biological activities and hold considerable potential for application as functional food components and therapeutic agents. These compounds possess various medicinal properties, including anti-inflammatory, antioxidant, anticancer and cardioprotective effects. While short-term use may have limited impact on human health, consistent intake or controlled pharmaceutical administration is considered important for achieving measurable health effects [52]. Isolated marine bioactives, on the other hand, may be concentrated, purified and structurally optimized for specific therapeutic targets, thereby moving beyond the functional food or nutraceutical domain into pharmaceutical development.

Beyond fully approved pharmaceuticals, marine-derived bioactive compounds are increasingly employed in clinical and quasi-clinical settings in the form of high-dose standardized extracts, peptide preparations, and medical nutrition formulations. These applications often involve concentrated or purified fractions derived from marine organisms, administered at doses substantially higher than those achievable through normal dietary intake. Marine-derived bioactive peptides and protein hydrolysates have been investigated for their antihypertensive, anti-inflammatory, and metabolic regulatory properties in human and preclinical studies [53]. In addition, reviews have highlighted the potential of marine peptides, particularly angiotensin-converting enzyme (ACE) inhibitory peptides derived from fish muscle and collagen hydrolysates, as functional components that may support cardiovascular health and serve as leads for pharmaceutical development [54].

**Figure 2:**
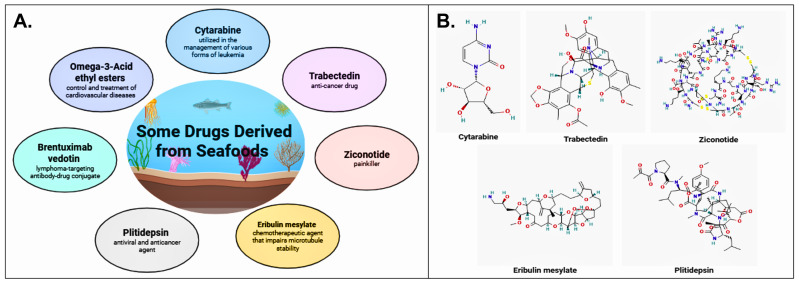
(**A**) Representative bioactive compounds and clinically approved drugs derived from seafood; (**B**) chemical structures of selected clinically approved drugs (generated using BioRender, https://www.biorender.com/).

Several compounds originally isolated from marine organisms have successfully progressed through clinical development and are currently approved for medical use. Such agents do not represent functional seafoods per se; rather, they exemplify the pharmaceutical translation of marine bioactive compounds following isolation, structural modification and regulatory approval. These include cytarabine (Cytosar-U^®^) for the treatment of leukemia, trabectedin (Yondelis^®^) as an anticancer agent, ziconotide (Prialt^®^) for the management of severe chronic pain, eribulin mesylate (Halaven^®^) as a chemotherapeutic agent affecting microtubule dynamics, plitidepsin (Aplidin^®^) with antiviral and anticancer activity, brentuximab vedotin (Adcetris^®^) as a lymphoma-targeting antibody–drug conjugate and omega-3-acid ethyl esters (Lovaza^®^) for the control and treatment of cardiovascular diseases [55]. Examples like these illustrate the translational trajectory of marine-derived bioactive compounds into regulated pharmaceutical products, while still remaining conceptually distinct from functional seafoods consumed as part of the diet (Figure 2).

### 4.1. Anticancer Marine Bioactive Compounds

Cytarabine, also known as cytosine arabinoside, was the first commercially available drug developed from marine compounds and was approved for clinical use by the Food and Drug Administration (FDA) in 1969. It remains a cornerstone chemotherapeutic agent in the treatment of hematological malignancies, particularly acute myeloid leukemia and non-Hodgkin’s lymphoma [56]. Cytarabine was originally isolated from the marine sponge *Tethya crypta* and is converted intracellularly to its active metabolite, cytarabine triphosphate, through phosphorylation catalyzed by deoxycytidine kinase. This active form competes with endogenous cytidine and becomes incorporated into DNA, where the arabinose sugar moiety interferes with DNA polymerase activity and effectively halts DNA synthesis [57].

In addition to inhibiting DNA polymerase, cytosine arabinoside is incorporated into both DNA and RNA, resulting in impaired nucleic acid synthesis and subsequent cytotoxicity. The antineoplastic activity of cytarabine is cell-cycle-specific, preferentially targeting rapidly dividing cells by preventing progression from the G1 phase to the S phase, ultimately leading to apoptosis [58]. Cytarabine is administered via injection, including intravenous infusion, intrathecal administration or subcutaneous injection, and undergoes hepatic metabolism. Common adverse effects include hematological toxicity, such as leukopenia, thrombocytopenia, anemia and bone marrow suppression, as well as gastrointestinal symptoms including nausea, vomiting, diarrhea and abdominal pain. For induction therapy in acute non-lymphocytic leukemia, cytarabine is typically administered at a dose of 100 mg/m^2^ either as a continuous intravenous infusion over 7 days or as an intravenous dose every 12 h for 7 days in combination with other anticancer agents [59].

Trabectedin, a marine-derived alkaloid originally isolated from the tunicate *Ecteinascidia turbinata*, received authorization from the European Medicines Agency (EMA) in 2007 for the treatment of advanced soft tissue sarcomas [60]. In 2009, it was approved in the European Union for use in combination with pegylated liposomal doxorubicin in patients with recurrent platinum-sensitive ovarian cancer. Subsequently, in 2015, the U.S. FDA approved trabectedin for patients with unresectable or metastatic liposarcoma or leiomyosarcoma previously treated with anthracycline-based chemotherapy. In addition to these indications, trabectedin has demonstrated cytotoxic activity against breast cancer cells in experimental studies [61].

Trabectedin exhibits a complex and pleiotropic mechanism of action. Its most extensively characterized activity involves binding to the minor groove of DNA, which leads to transcriptional interference and disruption of DNA repair pathways, resulting in accumulation of DNA damage and cell cycle arrest [62]. Beyond its direct cytotoxic effects on tumor cells, trabectedin also exerts immunomodulatory activity by selectively targeting monocytes and tumor-associated macrophages. This selective depletion suppresses the production of pro-inflammatory mediators within the tumor microenvironment, thereby contributing to its antitumor efficacy through both direct and indirect mechanisms [63,64].

Eribulin mesylate (E7389), marketed under the trade name Halaven^®^, is a synthetic analog of halichondrin B, a marine-derived natural product originally isolated from the sponge *Halichondria okadai*. It belongs to the halichondrin class of antineoplastic agents and functions as a non-taxane inhibitor of microtubule dynamics. Unlike other tubulin-targeting agents, eribulin selectively inhibits microtubule growth without affecting depolymerization, leading to the sequestration of tubulin into non-functional aggregates. This disruption of microtubule dynamics ultimately results in mitotic blockade and apoptosis in cancer cells [65].

In contrast to conventional microtubule-targeting agents, such as taxanes and vinca alkaloids, eribulin exhibits a distinct mechanism of action and has demonstrated reduced neurotoxicity while retaining activity in paclitaxel-resistant cancer cell lines carrying β-tubulin mutations. Clinically, eribulin mesylate is administered intravenously and was approved by the U.S. Food and Drug Administration in 2010 for the treatment of patients with locally advanced or metastatic breast cancer who had previously received at least two chemotherapy regimens including an anthracycline and a taxane [66].

### 4.2. Analgesic Marine Bioactive Compounds

Ziconotide is a synthetic analog of ω-conotoxin MVIIA originally isolated from the venom of the marine snail *Conus magus*. It is a 25-amino-acid peptide containing six cysteine residues that form three disulfide bridges, which are essential for maintaining its stable tertiary structure and biological activity. Ziconotide selectively inhibits N-type voltage-gated calcium channels and displays minimal interaction with other ion channels or with cholinergic, monoaminergic or peptidergic receptor systems [67].

Ziconotide exerts its analgesic effects by selectively binding to N-type voltage-gated calcium channels located on primary nociceptive afferent neurons in the superficial laminae of the dorsal horn of the spinal cord. These calcium channels function as key regulators of neurotransmitter release at synapses involved in nociceptive processing. Inhibition of channel activity suppresses calcium influx into presynaptic terminals thereby preventing the release of excitatory neurotransmitters, such as glutamate and substance P, which play a central role in nociceptive signaling and central sensitization. As a result, synaptic transmission between primary afferent neurons and second-order neurons in the spinal cord is disrupted leading to reduced neuronal excitability and attenuation of pain perception [68]. Due to its relatively large and hydrophilic molecular structure, ziconotide is unable to cross the blood–brain barrier. Consequently, intrathecal administration is required to achieve therapeutic concentrations at spinal targets. The FDA first authorized ziconotide in 2000, with full approval for the management of severe chronic pain granted in 2004, followed by EMA approval in 2005 [69].

### 4.3. Antiviral Marine Bioactive Compounds and Translational Therapeutic Applications

Plitidepsin (Aplidin^®^) is a cyclic depsipeptide originally isolated from the marine tunicate *Aplidium albicans* and is structurally related to didemnin B. It has garnered significant interest due to its potent anticancer and antiviral activities, primarily through its interaction with eukaryotic translation elongation factor 1 alpha (eEF1A), a key protein involved in the elongation phase of mRNA translation [70].

Plitidepsin exhibits high affinity for eEF1A2 (KD ≈ 80 nM), leading to disruption of protein synthesis in both malignant and virus-infected cells. Plitidepsin has shown therapeutic potential in several cancer types, including multiple myeloma, leukemia and breast cancer, and received regulatory approval in Australia in 2018 for the treatment of multiple myeloma. In addition to its anticancer activity, recent studies have demonstrated that plitidepsin also possesses broad-spectrum antiviral activity, including potent inhibition of SARS-CoV-2 replication, with an IC_90_ of 0.88 nM, in cell-based experimental systems. This in vitro potency reflects drug-development research rather than dietary exposure and does not imply achievable concentrations through functional seafood consumption. These antiviral effects are associated not only with inhibition of eEF1A-dependent translation, but also with suppression of double-membrane vesicle (DMV) biogenesis, essential for coronavirus replication [71]. Further mechanistic studies have shown that plitidepsin promotes degradation of eEF1A and inhibits both viral and host protein synthesis. In addition, induction of eIF2α phosphorylation has been reported, suggesting broader regulatory effects on translational initiation pathways [71]. Together, these findings highlight plitidepsin’s therapeutic potential as a dual-acting agent in oncology and infectious disease settings [72]. A summary of clinically approved seafood-derived compounds, their mechanisms of action, routes of administration and therapeutic applications is presented in Table 2.

## 5. Functional Seafoods and Pharmacological Mechanisms of Action

**Figure 3:**
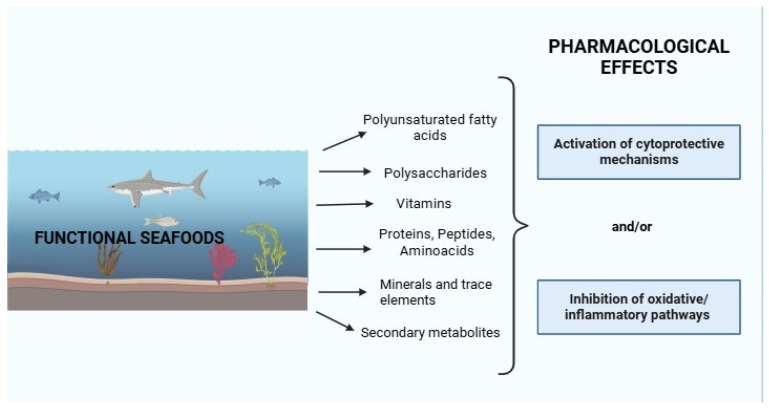
Molecular mechanisms underlying the pharmacological effects of the components of functional seafoods (generated using BioRender).

Since functional seafood products are increasingly used in the health industry, it is of critical importance to understand the pharmacological mechanisms underlying their therapeutic effects. Although research in this field has expanded in recent years, the mechanisms of action of many functional seafood-derived compounds remain insufficiently clarified. Further in-depth investigations are therefore required [35]. According to available experimental and clinical studies, functional seafood products generally exert their pharmacological effects through endogenous bioactive components, such as polysaccharides, vitamins, proteins, peptides, amino acids and polyunsaturated fatty acids. These bioactive molecules display a broad spectrum of pharmacological effects, encompassing antioxidant, anti-inflammatory and anticancer actions (Figure 3) [73].

Some functional seafood products whose pharmacological mechanisms of action have been elucidated, together with details of the studies conducted on these products, are described below.

### 5.1. Anti-Inflammatory and Immunomodulatory Mechanisms

It is widely reported that omega-3 fatty acids, which are polyunsaturated fatty acids found in seafood, exert beneficial effects on cardiovascular diseases. The synthesis of information derived from basic and clinical translational research studies indicated that omega-3 fatty acids derived from seafood may protect against heart failure through multiple mechanisms, including: (i) the inactivation of inflammatory mediators such as interleukin-1 (IL-1), interleukin-6 (IL-6) and nuclear factor kappa B (NF-κB), primarily demonstrated in experimental (cellular and animal) models with attenuation of myocardial inflammatory signaling, (ii) enhancement of nitric oxide release from vascular endothelial cells, shown in endothelial cell systems and reflected in improved endothelial function (e.g., flow-mediated dilation) in selected human supplementation studies, (iii) reduction in sympathetic nervous system activity, observed in clinical supplementation trials through decreases in circulating norepinephrine levels, (iv) lowering of blood pressure, documented in randomized controlled trials assessing n-3 PUFA supplementation, and (v) decreased calcium influx into vascular smooth muscle cells, reported in preclinical vascular reactivity models [74]. The mechanistic pathways described above reflect the current understanding of molecular interactions reported in the literature; however, the relative contribution of each pathway in human cardiovascular disease, particularly in the context of dietary intake levels and bioavailability constraints, requires further clarification. It is also noted that clinical evidence in heart failure remains heterogeneous and randomized controlled trials assessing dietary fish or n-3 PUFA intake for primary prevention of heart failure are lacking. Furthermore, bioavailability and clinical responses may vary depending on formulation and absorption conditions [74].

In addition to polyunsaturated fatty acids, several marine-derived proteins and secondary metabolites have also been investigated for their immunomodulatory and anti-inflammatory properties. For instance, the high-quality protein found in large amounts in sea cucumber has been shown in experimental animal feeding studies to reduce serum triglyceride levels. This protein is rich in glutamic acid, arginine and glycine. Among these amino acids, glycine has been documented to stimulate production and release of interleukin-2 (IL-2) and B-cell antibody and thereby enhance phagocytosis in experimental systems, glutamic acid and glycine are essential components for glutathione synthesis which has been associated with activation and proliferation of natural killer cells in vitro, and arginine can enhance cell immunity by promoting activation and proliferation of T-cells in model studies [6].

Similarly, bioactive metabolites from marine algae have been reported to influence inflammatory signaling pathways in experimental models. Rani et al. reported that secondary metabolites derived from *Gracilaria* species, a type of red algae, exhibit antioxidant, anti-inflammatory and anticancer activities in predominantly in vitro and preclinical experimental models. These effects were primarily mediated through the suppression of oxidative stress and inflammatory responses, involving inhibition of the NF-κB and Janus kinase/signal transducer and activator of transcription (JAK/STAT) signaling pathways as well as the induction of apoptosis in cellular systems [75].

Additional evidence for anti-inflammatory activity has also been reported for glycoproteins isolated from edible brown algae. In a study by Rafiquzzaman et al., a glycoprotein from *Undaria pinnatifida* was investigated for anti-Alzheimer’s-relevant activity in in vitro enzymatic and primary neuronal cell models. The compound significantly inhibited acetylcholinesterase (AChE), butyrylcholinesterase (BChE), and β-secretase (BACE1), key enzymatic targets implicated in Alzheimer’s disease pathogenesis, in dose-dependent in vitro assays, and enhanced neuronal survival in primary rat hippocampal cultures without inducing cytotoxicity. Furthermore, in lipopolysaccharide-stimulated murine macrophage cells (RAW 264.7) and in cyclooxygenase enzyme assays, this glycoprotein reduced nitric oxide production and inhibited cyclooxygenase-1 (COX-1) and cyclooxygenase-2 (COX-2) enzyme activity, supporting its anti-inflammatory and antioxidant effects within controlled experimental systems [76].

### 5.2. Antioxidant and Cytoprotective Mechanisms

Primarily through antioxidant defense pathways, in vitro and in vivo experimental studies have demonstrated that polysaccharides derived from the seaweed *Enteromorpha prolifera* mitigate oxidative stress and inflammatory responses through modulation of the Nuclear Factor Erythroid 2–Related Factor 2 (Nrf2) and NF-κB signaling pathways, as shown by reductions in heat stress-induced splenic oxidative stress markers and suppression of NF-κB p65 signaling in a chicken (*Gallus gallus domesticus*) model of heat stress [77].

Comparable antioxidant and cytoprotective effects have been reported for protein extracts derived from other marine microorganisms. Bermejo-Bescós et al. demonstrated that protein extracts from *Spirulina platensis* exhibited antioxidant properties in SH-SY5Y human neuroblastoma cells exposed to iron-induced oxidative stress, by mitigating iron-induced oxidative damage and preserving the activity of cellular antioxidant enzymes including glutathione peroxidase (GPx) and glutathione reductase (GR), while also reducing lipid peroxidation levels [78].

In addition to specific bioactive molecules, the mineral composition of seafood may also contribute to antioxidant and metabolic homeostasis. Awuchi et al. noted that fish and other seafood contain essential minerals and trace elements, including calcium, sodium, iron and magnesium. Calcium plays a key role in bone and tooth formation, sodium is essential for neurotransmission and electrolyte balance, iron contributes to hemoglobin structure and magnesium regulates electrolyte homeostasis. In addition, minerals such as magnesium, zinc and iodine act as cofactors in various enzymatic reactions, based on established physiological roles in human metabolism [35].

Trace elements associated with marine proteins have also been investigated for their potential cytoprotective effects. In a study by Song et al., selenium-enriched proteins derived from *Spirulina platensis* were shown in a neuronal experimental model to inhibit neurotoxicity and apoptosis in hippocampal neurons. This selenium-enriched protein ameliorated mitochondrial dysfunction and suppressed DNA damage by reducing reactive oxygen species accumulation and stabilizing the expression of genes belonging to the Bcl-2 family in vitro [79].

Other lipid-soluble micronutrients present in marine organisms have similarly been associated with antioxidant mechanisms. Alpha-, beta- and gamma-tocopherol, homologs of vitamin E, were reported to modulate signal transduction and gene expression primarily in experimental cellular and membrane model systems, while also exerting potent antioxidant effects [80,81]. Similarly, Boominathan and Mahesh showed that beta-carotene, a precursor of vitamin A found in seaweeds, exhibits antioxidant activity, in experimental models of oxidative stress and carcinogenesis, thereby interfering with oxidative mechanisms implicated in tumor development [82].

### 5.3. Metabolic and Cellular Signaling Mechanisms

In an in vitro study examining the antioxidant and anticancer properties of phycocyanin, a biliprotein present in *Spirulina platensis,* its effects on modulating the phosphatidylinositol 3-kinase (PI3K), protein kinase B (Akt), mammalian target of rapamycin (mTOR), and Wnt pathways were examined. It was shown that treatment with c-phycocyanin (IC_50_ 100 µg/mL) for 48 h produced a cytotoxic effect, compared to diethylether extract (IC_50_ 630 µg/mL) and Paclitaxel (IC_50_ 10 µg/mL), which was used as a positive control. More specifically, c-phycocyanin induced apoptosis in MCF-7 breast cancer cell lines by downregulating the expression of the above key signaling molecules. In addition, phycocyanin activated caspase-8, caspase-9 and caspase-3, demonstrating a pronounced anticancer effect [83].

Beyond these signaling pathways, *Spirulina*-derived compounds have been investigated for their influence on tumor-related cellular signaling mechanisms. Chen et al. reported that *Spirulina* extract, phycocyanin and allophycocyanin inhibited the migration and peritoneal dissemination of endometrial cancer cells in a nude mouse xenograft model, by modulating the Transforming Growth Factor-β (TGF-β)/Mothers against the decapentaplegic homolog 4 (SMAD4) signaling pathway, with associated reductions in tumor cell migration and metastatic spread in vivo [84].

Other microalgal proteins have also been explored for their interaction with metabolic regulatory pathways. In a study conducted by Li et al., proteins derived from *Chlorella pyrenoidosa* were evaluated for their bioactive peptides targeting dipeptidyl peptidase-4 (DPP-IV) and ACE. In vitro enzymatic and cellular assays demonstrated ACE and DPP-IV inhibitory activity, suggesting a potential role in pathways relevant to cardiometabolic regulation. Peptic and tryptic hydrolysates inhibited ACE activity in vitro in a dose-dependent manner at concentrations of 1, 2.5 and 5 mg/mL, with peptic hydrolysates showing slightly higher activity. Both hydrolysates exhibited similar inhibitory effects at the cellular level and reduced DPP-IV activity at a concentration of 5 mg/mL in cell-based assays [85].

To provide an overview of the diverse mechanisms described above, a summary of selected bioactive compounds derived from seafood products, their pharmacological activities and proposed mechanisms of action is presented in Table 3.

In conclusion, functional seafood products have been reported to exhibit antioxidant, anti-inflammatory and anticancer properties mediated by a variety of bioactive compounds, including polyunsaturated fatty acids, proteins, peptides, amino acids, polysaccharides, vitamins and phenolic compounds. The molecular mechanisms underlying these pharmacological effects have been primarily characterized in experimental models and generally involve inhibition of oxidative and inflammatory pathways and/or activation of cytoprotective responses. Given the importance of these effects in experimental and translational research contexts, further research is required to clarify the molecular mechanisms associated with functional seafood products, particularly those whose pharmacological actions remain inadequately understood.

## 6. Evidence Linking Functional Seafood Consumption to Human Health Outcomes

Unless otherwise specified, the evidence discussed in this section refers to dietary intake of seafood or nutraceutical supplementation, rather than approved pharmaceutical agents.

Functional seafoods, as components of dietary patterns or nutraceutical supplementation, have attracted increasing attention in clinical nutrition due to their potential role in the prevention and management of various chronic diseases. Their potential clinical relevance is largely attributed to the presence of bioactive compounds such as omega-3 fatty acids, polysaccharides, peptides and polyphenols which have been reported to exert antioxidant, anti-inflammatory and immunomodulatory effects. However, interpretation of the clinical implications discussed below should consider variability in bioavailability and achievable intake levels under dietary conditions.

Evidence relating functional seafood consumption to human health outcomes spans multiple disease domains, including gastrointestinal disorders, cardiometabolic diseases, neurodegenerative conditions and cancer. In the following subsections, findings from epidemiological studies, human intervention trials and experimental mechanistic investigations are discussed according to the major disease areas in which marine bioactive compounds have been studied. Where relevant, distinctions are made between evidence derived from dietary intake, nutraceutical supplementation and experimental systems.

### 6.1. Gastrointestinal Diseases

Gastrointestinal tract (GI) diseases represent a major global health problem, significantly affecting quality of life. Dietary habits and lifestyle factors are widely recognized contributors to the development of gastrointestinal disorders. A balanced diet plays a critical role in maintaining normal GI function and has been associated in clinical and observational studies with reduced risk of disorders such as gastritis, ulcers and colitis. In this context, it has been reported that the inclusion of seafood and nuts rich in omega-3 fatty acids in the diet is associated with anti-inflammatory effects and with modulation of pathways implicated in chronic disease development [95]. It has also been documented that adherence to a Mediterranean-type diet and the regular consumption of vegetables, fruits and seafoods are associated with alterations in intestinal microbiota composition in human studies [96]. However, much of the mechanistic evidence discussed below derives primarily from experimental and preclinical studies, while human clinical data remain comparatively limited.

Polysaccharides, including sulfated polysaccharides such as fucoidan, alginate and chondroitin sulfate which are found in brown, red and green algae, have demonstrated antioxidant and anti-inflammatory activities in experimental and preclinical studies and have also been reported to exert protective effects on the gastrointestinal mucosa primarily in in vitro and animal models. These polysaccharides have been shown in experimental systems to modulate intestinal microbiota composition and contribute to the regulation of intestinal homeostasis. Studies have demonstrated that fucoidan sulfate can bind to *Helicobacter pylori* and inhibit its adhesion to gastrointestinal epithelial cells in cell-based adhesion assays. In addition, polysaccharides have been reported in animal and in vitro models to regulate intestinal flora and enhance intestinal barrier function. Furthermore, polyphenolic compounds present in seafood have been reported to exert antioxidant and anti-inflammatory effects and to display prebiotic activity within the intestinal microbiota [97].

Recently, increasing attention has also been directed towards polyphenolic compounds found in functional marine products. These polyphenols have been described in experimental studies as exhibiting prebiotic-like effects and multiple pharmacological activities [97]. Review analyses further indicate that sulfated polysaccharides such as fucoidan, alginate, and chondroitin sulfate found in brown, red, and green algae demonstrate antioxidant and anti-inflammatory activities primarily in experimental models and may protect the gastrointestinal mucosa, in cell and animal studies, by modulating the intestinal microbiota composition, regulating intestinal homeostasis and strengthening intestinal barrier function [97]. These studies also report that fucoidan sulfate can bind to *Helicobacter pylori* and inhibit its adhesion to gastrointestinal epithelial cells in cell-based assays [97].

Overall, the available findings indicate that several marine-derived nutrients and bioactive compounds may influence gastrointestinal physiology through antioxidant, anti-inflammatory and microbiota-related mechanisms. However, much of the supporting evidence currently derives from experimental and preclinical studies and further well-designed human investigations are required to clarify their clinical relevance.

### 6.2. Cardiometabolic Diseases

Cardiometabolic diseases encompass a spectrum of interrelated conditions including cardiovascular disease, hypertension, dyslipidaemia and type-2 diabetes, which share common metabolic, inflammatory and oxidative stress pathways. Compared with other disease domains discussed in this review, these disorders represent the area where the most substantial human observational and clinical evidence relating seafood-derived nutrients to health outcomes is currently available.

Cardiovascular disease (CVD) remains the leading cause of mortality worldwide, substantially impairing quality of life. According to statistical data, more than 17 million deaths were attributed to CVD in 2017 with projections suggesting this figure will rise to 23 million by 2030 [98]. In order to mitigate the prevalence of CVD, it is essential to assess not only established risk factors but also the underlying molecular mechanisms involved in disease development [99].

Increased oxidative stress plays a pivotal role in the pathogenesis of CVD, contributing to conditions such as myocardial infarction, heart failure and ischemia–reperfusion injury [100]. The overproduction of reactive oxygen species (ROS) reduces nitric oxide bioavailability leading to vasoconstriction and the subsequent development of arterial hypertension. Moreover, ROS disrupt calcium homeostasis in cardiac muscle, which may result in arrhythmias. Oxidative stress is also implicated in the formation and progression of atherosclerotic plaques [99].

Seafood, such as fish and shrimp, contain selenium (Se), which contributes to cellular redox balance. In a 90-day dietary selenium deficiency rat model, reduced selenium intake was associated with decreased Bcl-2 expression and increased cleaved caspase-3 and caspase-9 levels in myocardial tissue [101,102]. Furthermore, selenium has been demonstrated to influence potassium channel expression, mitochondrial function and STAT3 activity in primary cultured neonatal mouse cardiomyocytes exposed to low selenium concentrations [103]. In other studies, selenium supplementation in experimental rat models of ischemia–reperfusion injury was associated with increased myocardial STAT3 protein levels and reduced biochemical markers of tissue injury [104,105].

Omega-3 fatty acids found in microalgae have been associated in clinical and experimental studies with reductions in triglyceride levels, blood pressure and thrombotic markers [106]. In a randomized clinical intervention study in patients with type-2 diabetes, supplementation with purified eicosapentaenoic acid, one of the omega-3 fatty acids commonly found in seafood, at a dose of 1.8 g/day for 24 months, significantly reduced the progression of carotid intima–media thickness compared with standard therapy, supporting an anti-atherosclerotic effect in a high-risk population [107]. In addition, omega-3 fatty acids have been shown to modulate endothelial function and vascular inflammatory pathways through effects on nitric oxide bioavailability and inflammatory signaling, as summarized in mechanistic and translational studies [74].

Similarly, peptides, which are by-products of seafood, have demonstrated ACE inhibitory activity in experimental and preclinical studies, which is relevant to hypertension and other CVD [29]. In addition to algae-derived peptides, shellfish protein hydrolysates have also demonstrated ACE inhibitory activity and blood pressure–lowering effects in experimental animal models of hypertension. These effects have been attributed to short peptide sequences enriched in hydrophobic and aromatic amino acids, which enhance binding affinity to the ACE active site and may improve vascular function through modulation of nitric oxide bioavailability and inflammatory mediators [108].

Furthermore, vitamin D, found in seafood and other foods, has been associated in observational and mechanistic studies with modulation of cardiovascular structure and function, rather than directly preventing CVD [109]. Astaxanthin, a carotenoid found in seafood and microalgae, has been evaluated in clinical studies of patients with heart failure, where improvements in selected cardiac function parameters were reported [110].

Kadokura et al. reported that rats fed crab-flavored seafood exhibited improvements in CVD-related serum biomarkers [111]. In addition to such animal-based findings, several bioactive compounds derived from marine algae have also been associated with cardioprotective effects. Fucoxanthin, a carotenoid found in brown macroalgae, has been reported to attenuate the development of CVD in mice subjected to a high-fat diet through multiple mechanisms, including reduction in oxidative stress, inhibition of inflammatory processes and suppression of vasoconstrictor activity [112]. Similarly, zeaxanthin, a carotenoid present in microalgae and algae, was orally administered at a level of 250 µg/kg in rats with induced cardiac dysfunction, and was reported to contribute to CVD prevention by improving endothelial function and exerting antioxidant and anti-inflammatory effects [113]. Notably, the EU Commission has approved a use level of 2 mg zeaxanthin per day [114].

Beyond carotenoids, certain marine-derived polysaccharides are also being investigated in the context of cardiovascular protection. For instance, alginate oligosaccharides found in brown seaweeds have demonstrated anti-inflammatory activity and reductions in vascular inflammation and blood pressure primarily in experimental and preclinical models and have been reported to improve vascular function in these settings [113]. In addition, phlorotannins derived from brown seaweeds have been reported to exert antioxidant and anti-inflammatory effects in cellular and animal systems, suggesting potential cardioprotective relevance; however, human intervention data remain limited [115]. A summary of bioactive components derived from functional seafoods and their reported effects on the cardiovascular system is presented in Table 4.

Collectively, available evidence suggests that several marine-derived nutrients and bioactive compounds may contribute to cardiometabolic health through modulation of lipid metabolism, vascular function, oxidative stress and inflammatory pathways, depending on compound class and study design.

### 6.3. Neurodegenerative Diseases

Neurodegenerative diseases are progressive disorders characterized by neuronal damage and degeneration of myelin sheaths. Oxidative stress and mitochondrial dysfunction play central roles in the development and progression of these disorders, while neuroinflammation also contributes significantly to their pathophysiology [52,116]. In such conditions, elevated ROS levels compromise cellular integrity, while mitochondrial metabolic dysfunction leads to structural and functional neuronal damage. A self-perpetuating cycle between oxidative stress and mitochondrial dysfunction further promotes neuronal cell death and ultimately results in neuronal loss. Overall, oxidative damage, mitochondrial impairment and neuronal loss represent interconnected hallmarks of the pathophysiological mechanisms underlying neurodegenerative diseases [52,117]. Nevertheless, the majority of studies examining marine-derived bioactive compounds in this context originate from cellular and animal models, while consistent clinical evidence in human populations remains limited.

**Figure 4:**
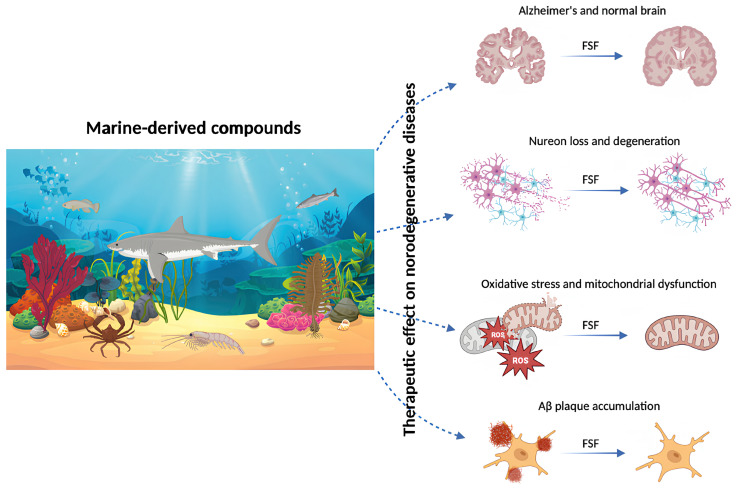
Marine-derived compounds and their potential effects on neurodegenerative diseases (FSF: Functional Seafoods) (generated using BioRender).

Bioactive compounds derived from marine organisms exhibit anti-inflammatory, antioxidant and neuroprotective properties and have demonstrated therapeutic potential primarily in experimental and preclinical studies of neurodegenerative disorders, such as Parkinson’s disease (PD) and Alzheimer’s disease (AD) (Figure 4) [118]. These marine-derived compounds may protect neuronal cells in the brain by attenuating oxidative stress, a key contributor to neurodegeneration. In particular, omega-3 fatty acids, peptides and polysaccharides derived from seafood have been reported to improve brain function and support neuronal health.

Omega-3 fatty acids may decrease neuroinflammation in AD by promoting the elimination of amyloid beta plaques from the brain. These findings are principally derived from experimental and preclinical models and translation of such effects to consistent clinical benefit in human populations remains under active investigation [52,118]. Furthermore, some peptides derived from seafood have been shown in cellular models to suppress oxidative damage in neuroblastoma cells by reducing apoptosis and intracellular ROS production. Such peptides have also been reported in animal models, including rodents and other experimental organisms, to alleviate cognitive impairments and improve learning and memory performance [119].

Porphyran, a polysaccharide known for its antioxidant properties, has demonstrated neuroprotective effects against amyloid beta-induced neurotoxicity in a mouse model of AD following intracerebroventricular Aβ1–40 administration. Studies indicate that porphyran improves amyloid beta-associated learning and memory impairments in cortical and hippocampal regions in this experimental model. In addition, it contributes to the preservation of neurological functions by regulating the activity of acetylcholinesterase and choline acetyltransferase, enzymes which play critical roles in synaptic transmission, as demonstrated in preclinical in vivo studies [120].

Beyond porphyrans and omega-3 fatty acids, additional marine-derived natural products have attracted interest for their potential benefits in neurodegenerative disorders. For example, fucoidan isolated from brown seaweeds has been shown to improve motor function in preclinical PD models and to protect dopaminergic neurons in vivo. Furthermore, cerebrosides derived from sea cucumber have been reported to enhance cognitive function and regulate synaptic plasticity in experimental AD models. Marine-derived pigments, such as carotenoids, may also delay neurodegenerative processes in cellular and animal systems by protecting neurons against oxidative stress through their antioxidant and anti-inflammatory properties [118]. A summary of marine-derived bioactive compounds associated with neurodegenerative diseases and their reported neuroprotective effects is presented in Table 5.

Recent research suggests that omega-3 fatty acids may play a role in alleviating symptoms of Huntington’s disease (HD). The anti-inflammatory, antioxidant and neuroprotective properties of these fatty acids indicate their potential relevance in mitigating neurodegenerative mechanisms associated with HD, primarily based on experimental and preclinical studies. However, studies directly examining the effects of marine-derived compounds on HD remain limited and clinical evidence is currently inadequate [130]. Similarly, research on functional seafoods in other neurodegenerative disorders, such as prion diseases, spinocerebellar ataxias, spinal muscular atrophy and motor neuron diseases, is currently insufficient.

In conclusion, functional seafood products may be considered sources of bioactive agents with potential relevance for therapeutic intervention in neurodegenerative diseases, particularly AD and PD, due to their antioxidant, anti-inflammatory and neuroprotective properties. Their ability to regulate synaptic plasticity, alleviate oxidative stress and modulate neurotransmitter balance has been demonstrated primarily in cellular and animal models, suggesting a multifaceted mechanistic relevance for a protective role in the central nervous system [118].

### 6.4. Cancer

Cancer is a serious disease characterized by rapid and abnormal cell growth, with the ability to spread to other parts of the body, making it both complex and life-threatening. While traditional approaches such as surgery, chemotherapy and radiation therapy remain fundamental in cancer treatment, their severe side effects have driven the search for less toxic and more effective alternatives. In this respect, developments in biotechnology have facilitated the advancement of innovative treatment strategies, contributing to improved treatment outcomes and increased survival rates in certain cancer types [131,132,133]. As a result, natural substances are increasingly being explored as promising candidates for the development of new anticancer therapies. At present, however, most evidence supporting anticancer activities of marine-derived compounds derives from cellular and experimental tumor models, with only a limited number of compounds progressing to clinical evaluation.

**Figure 5:**
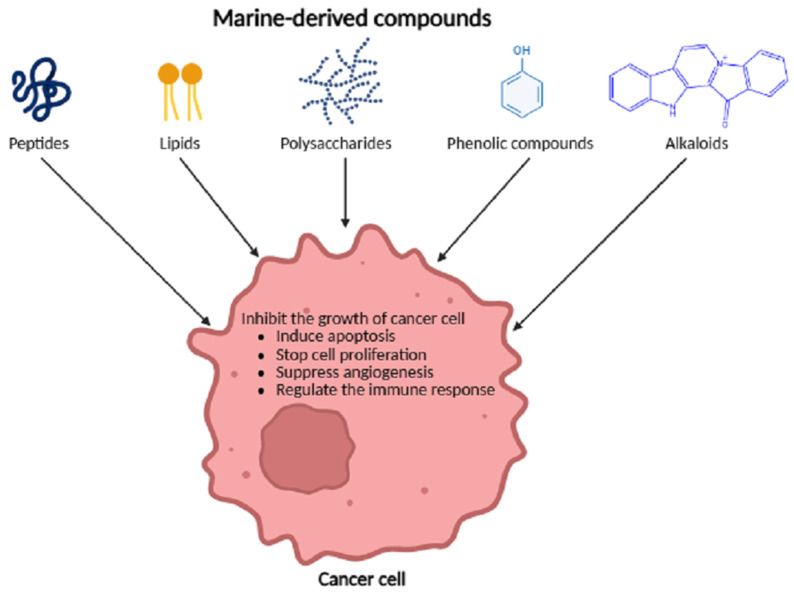
Biological effects of marine-derived compounds on cancer cells (generated using BioRender).

Among these natural sources, marine-derived compounds have attracted considerable interest due to their potential antitumor properties. Numerous studies have demonstrated that bioactive compounds originating from marine organisms can inhibit cancer cell proliferation, induce apoptosis and suppress tumor progression in cellular systems and experimental tumor models [134,135]. These compounds encompass a broad spectrum of chemical classes, including polysaccharides, phenolic compounds, alkaloids and peptides (Table 6) [3]. For example, fucoidan, a sulfated polysaccharide extracted from brown algae, has exhibited anticancer activity against breast, lung and colon cancer cells in vitro and in preclinical animal models by inhibiting angiogenesis, modulating immune responses and inducing apoptosis (Figure 5) [136].

Several bioactive metabolites, obtained from marine organisms, have already been developed into clinically approved anticancer drugs, demonstrating significant efficacy in the prevention and therapeutic intervention of cancer. Eribulin mesylate, cytarabine, trabectedin and brentuximab vedotin are among the marine-derived drugs currently applied in clinical settings for the treatment of acute leukemia, metastatic breast cancer, T-cell lymphoma and ovarian cancer, respectively [134].

Evidently, natural compounds derived from marine sources have contributed to the development of several anticancer agents and continue to stimulate research aimed at the discovery of novel therapeutic candidates. Numerous marine-derived compounds demonstrate antiproliferative and pro-apoptotic activities in cellular systems and experimental tumor models and are currently being investigated in preclinical and clinical studies. Nevertheless, translation of these findings into clinically validated therapies remains limited and requires further translational and clinical investigation.

Overall, the available evidence suggests that marine-derived nutrients and bioactive compounds may influence multiple aspects of human health across diverse disease domains. While numerous mechanistic and experimental studies support potential biological activities of marine bioactive compounds, the strength of evidence varies substantially between compound classes and study designs. Consequently, further well-designed human intervention studies are required to establish causal relationships and to define realistic exposure and bioavailability conditions associated with clinically relevant health outcomes

## 7. Considerations on Exposure, Bioavailability and Translational Relevance

Although numerous marine-derived bioactive compounds demonstrate promising mechanistic effects in cellular and animal models, a large proportion of the available evidence derives from such experimental systems rather than controlled human studies [134,135]. Consequently, their clinical relevance depends on achievable exposure levels through diet or supplementation, as well as their absorption and metabolic fate in the human body. Mechanistic findings discussed in previous sections should therefore be interpreted in the context of realistic exposure conditions that can be achieved through dietary intake or nutraceutical supplementation.

For example, omega-3 fatty acids derived from seafood are among the few marine bioactives for which human pharmacokinetic and intervention data are well established [74], with plasma concentrations typically reaching the low micromolar range following dietary intake or supplementation, as reported in clinical studies evaluating vascular endpoints. This alignment between experimentally observed biological effects and achievable exposure levels has contributed to the relatively strong translational evidence supporting the cardiovascular and metabolic benefits of marine-derived omega-3 fatty acids [74]. In contrast, some mechanistic investigations of other marine-derived compounds described in Section 5 and Section 6 rely on in vitro systems, in which antioxidant, anti-inflammatory or antiproliferative effects of marine polysaccharides, polyphenols or peptides are observed at concentrations that may substantially exceed physiologically attainable systemic levels [118,134,135,136]. This discrepancy between experimentally applied concentrations and physiologically attainable exposure levels restricts the direct translation of some experimental findings to human dietary exposure scenarios. Accordingly, interpretation of mechanistic findings requires consideration of compound bioavailability under realistic exposure conditions, which represents a key determinant of whether experimentally observed biological activities may actually occur in humans.

In addition, several classes of marine-derived compounds, including high-molecular-weight polysaccharides such as fucoidan or porphyran, may undergo limited intestinal absorption and substantial biotransformation by gut microbiota, potentially modifying their bioactivity profile in vivo, as discussed in studies addressing their preclinical effects [103,120]. These characteristics suggest that some biological effects attributed to marine polysaccharides may arise primarily from interactions within the gastrointestinal environment or from metabolites generated during microbial fermentation rather than from systemic exposure to the intact compounds. Indeed, microbial fermentation processes may generate lower molecular weight metabolites and short-chain fatty acids that can influence or modulate biological responses in the gastrointestinal tract and potentially systemically [118].

Similarly, bioactive peptides may be subject to gastrointestinal digestion before reaching systemic circulation, and lipophilic compounds such as carotenoids depend on dietary matrix and lipid co-ingestion for optimal absorption [118]. These pharmacokinetic constraints define important boundaries for extrapolating biological activities observed in controlled experimental systems to human dietary exposure scenarios. While preclinical models provide valuable insights into molecular pathways, further studies addressing dose–response relationships, pharmacokinetics and controlled human interventions are required to better define clinically relevant exposure ranges and their potential translational applicability and to establish safe use levels for maximum efficacy of marine-derived bioactive compounds [10].

## 8. Challenges and Future Perspectives

Despite the growing interest in functional seafoods, significant knowledge gaps remain regarding their health benefits and potential risks. Research in this field is still fragmented and often lacks interdisciplinary integration [142]. Overall, further studies are required to elucidate critical issues related to the bioavailability and efficacy of seafood-derived bioactive compounds and their long-term health effects [143]. In the absence of sufficiently robust evidence supporting the use of certain marine bioactive compounds for human consumption, some potentially valuable resources may remain underutilized. Regulatory assessment therefore becomes challenging and complicated and may considerably vary depending on the region of food production and marketing [8,144].

In addition to regulatory heterogeneity, a more explicit risk–benefit evaluation is required when translating marine bioactives into functional products. Seafood may contribute to exposure to environmental contaminants including methylmercury, cadmium, dioxins/dioxin-like PCBs and PFAS, depending on species, trophic level and geographic origin, as reflected in contemporary risk assessments by food-safety authorities [145,146,147,148]. Consumer awareness of chemical contaminants in fish and seafood remains variable across EU populations, underscoring the importance of risk communication and monitoring in functional seafood contexts [149]. While habitual seafood consumption within recommended dietary patterns is generally associated with net health benefit at population-level assessments [150,151], safety considerations may differ when concentrated extracts or purified compounds are consumed at levels exceeding typical dietary exposure, since long-term safety data for high-dose formulations of marine origin remain limited in several cases. Furthermore, allergenicity, particularly in relation to shellfish-derived proteins, represents a clinically relevant constraint for susceptible individuals [152]. In this context, robust quality control, batch-to-batch standardization and contaminant monitoring are essential to ensure product safety and to prevent disproportionate efficacy claims. Clear distinction between food-based intake and pharmacological dosing paradigms is therefore essential to avoid disproportionate efficacy claims or inappropriate extrapolation of safety expectations.

Furthermore, producers and consumers are often confronted with inconsistent regulatory frameworks related to health claims, labelling and safety requirements. For example, in the European Union, health claims for omega-3 fatty acids and bioactive peptides must comply with Regulation (EC) No 1924/2006 and require prior scientific substantiation and EFSA approval [153]. In the United States, on the other hand, structure–function claims may be used under the Dietary Supplement Health and Education Act (DSHEA) without pre-market authorization, provided that appropriate disclaimers are included [154]. Such differences create uncertainty for international commercialization. Similarly, maximum permitted levels of contaminants, such as heavy metals (e.g., mercury in fish oils) and marine biotoxins, differ between jurisdictions, which may create additional compliance requirements for exporters [150]. The stringent requirements imposed by regulatory agencies are often difficult to interpret and implement, particularly for small and medium-sized enterprises [155].

Moreover, scaling up the production of functional seafoods to meet global demand presents significant bottlenecks. Key constraints include limited and seasonal availability of raw marine biomass, variability in bioactive compound concentrations due to environmental conditions, and challenges in ensuring batch-to-batch consistency. Processing technologies such as enzymatic hydrolysis, membrane filtration and supercritical fluid extraction require substantial capital investment and technical expertise, which may not be readily accessible to all producers. Cold-chain logistics, especially for highly perishable marine products and microalgal biomass, further increase operational costs and energy consumption. Maintaining strict quality and safety standards at industrial scale, while preserving bioactivity, also remains technically demanding [156].

Successful technology transfer from research to market also requires standardization of analytical methods used to evaluate sensory properties, consumer acceptability and product quality, as well as well-designed clinical trials capable of substantiating health claims under realistic dietary exposure conditions. In parallel, the environmental impact of large-scale aquaculture operations must be carefully evaluated to ensure sustainability. Emerging biotechnological advances hold significant potential to shape the future of functional seafoods. Novel genetic engineering approaches, including selective breeding and precision aquaculture, may enhance sustainability, efficiency and resilience in seafood production systems. In this context, the development of disease-resistant fish species and the replacement of antibiotics with probiotics and vaccines may contribute to improved fish health. Functional ingredients derived from fish processing by-products also represent a promising research direction. For example, the isoelectric solubilization and precipitation method has been successfully applied for the extraction of valuable proteins and omega-3 fatty acids from fish waste [157]. These ingredients may subsequently be incorporated into functional foods, nutraceuticals and superfood formulations, aimed at improving cardiovascular health and reducing inflammation.

Sustainability remains a critical factor for the future development of functional seafoods. Advances in aquaculture technologies, such as recirculating aquaculture systems and the integration of renewable energy inputs, may contribute to reducing the environmental footprint of production. In addition, the incorporation of blockchain technology into seafood supply chains may enhance traceability, transparency and consumer trust [156]. However, the economic feasibility and scalability of these technologies require further evaluation to ensure that sustainability goals align with market realities.

## 9. Conclusions

This review has sought to position functional seafoods within a structured pharmacological and translational context rather than treating them solely as nutritional commodities. By integrating mechanistic evidence, clinical validation status and regulatory considerations, it provides a more explicit framework for distinguishing compounds supported primarily by experimental findings from those with stronger levels of clinical or translational evidence.

Functional seafoods represent a promising area in nutrition and preventive medicine, providing a wide range of bioactive components that have been associated with various biological activities. These naturally derived compounds, including omega-3 fatty acids, marine peptides, polysaccharides and antioxidants, have been reported to exhibit anti-inflammatory, cardioprotective, neuroprotective and anticancer activities in experimental and, in some cases, clinical studies. Their therapeutic potential extends to the prevention and management of chronic diseases, such as cardiovascular disorders, neurological conditions and metabolic syndromes, with varying levels of preclinical and clinical validation.

Advances in biotechnology and sustainable aquaculture practices have facilitated improved extraction, stabilization and characterization of marine-derived bioactive compounds, supporting their integration into modern dietary and nutraceutical approaches. Despite this potential, several challenges remain, including the need for standardized extraction and characterization methods, more consistent evidence from well-designed human studies and scalable production systems that are compatible with the preservation of marine ecosystems.

Future research should focus on elucidating the molecular mechanisms underlying the biological activities of marine bioactives, optimizing delivery strategies and addressing concerns related to long-term safety and allergenicity. In addition, greater attention to dose–response relationships and bioavailability under real-world dietary conditions will be essential to strengthen evidence-based health claims. Effective collaboration among researchers, industry stakeholders and policymakers will be essential for the establishment of robust regulatory frameworks and the sustainable exploitation of marine resources. With increasing consumer awareness and scientific support, functional seafoods are poised to play an important role in personalized nutrition and public health strategies, bridging the gap between nutrition and therapeutics, while promoting environmental stewardship for future generations.

## Figures and Tables

**Table 1:** Essential seafood: nutritional value and associated health benefits.

Seafood	Key Nutrients	Associated Health Benefits	Reference
Salmon	Omega-3 fatty acids, Vitamin D, Protein, Vitamin B12	Supports cardiovascular health, contributes to mood regulation, reduces inflammatory markers and supports cognitive function; particularly relevant for cardiovascular health, cognitive function and mood support	[20]
Sardines	Vitamin D, Calcium, Vitamin B12, Omega-3 fatty acids	Supports bone health, contributes to cardiovascular health and may reduce inflammation and support cognitive wellbeing; particularly relevant for bone health, cardiovascular health and inflammation	[36]
Mackerel	Protein, Omega-3 fatty acids, Vitamin D, Vitamin B12	Associated with reduced cardiovascular risk, anti-inflammatory effects and support of brain function; particularly relevant for brain health, inflammation and cardiovascular health	[37]
Tuna (Albacore)	Omega-3 fatty acids, Selenium, Protein, Vitamin B6	Supports cardiovascular health, muscle maintenance and immune function; particularly relevant for cardiovascular health, muscle health and immune support	[38]
Oysters	Zinc, Vitamin B12, Protein, Omega-3 fatty acids	Supports immune function and reproductive and skin health; particularly relevant for immune support, skin health and fertility	[39]
Shrimp	Protein, Omega-3 fatty acids, Vitamin D, Selenium	Supports muscle repair, cardiovascular health and immune function; particularly relevant for muscle health, cardiovascular health and immune support	[40]
Clams	Iron, Vitamin B12, Protein, Omega-3 fatty acids	Supports red blood cell production, cardiovascular health and immune function; particularly relevant for iron deficiency, cardiovascular health and immune support	[41]
Anchovies	Omega-3 fatty acids, Protein, Calcium, Vitamin A	Supports cardiovascular health, contributes to reduced inflammation and supports bone health; particularly relevant for bone health, cardiovascular health and inflammation	[42]
Cod	Protein, Vitamin B12, Iodine, Omega-3 fatty acids	Supports thyroid function, cardiovascular health and inflammatory balance; particularly relevant for thyroid health, cardiovascular health and inflammation	[43]
Marine Collagen (from fish)	Type I collagen, Omega-3 fatty acids, amino acids (glycine, proline)	Supports skin elasticity, joint and connective tissue health, gut health and bone integrity; particularly relevant for skin health, muscle and joint health, gut health and bone support	[44]

**Table 2:** Routes of administration, dosing, mechanism of action and side effects of some clinically approved seafood-derived drugs.

Seafood-Derived Drug	Routes of Administration	Dosing	Mechanism of Action	Side Effects	References
Cytarabine	Injectable (intravenous infusion, intrathecal or subcutaneous)	100 mg/m^2^ either as a daily continuous intravenous infusion (days 1–7) or as an intravenous dose administered every 12 h (days 1–7), alongside other anticancer medications	Activated by deoxycytidine kinase to cytarabine triphosphate; inhibits DNA polymerase; incorporates into DNA and RNA; arrests the cell cycle at the G1–S phase; induces cytotoxicity in rapidly dividing cancer cells	Bone marrow suppression-associated leukopenia, thrombocytopenia, and anemia, accompanied by nausea, vomiting, diarrhea, and abdominal pain	[57,58,59]
Trabectedin	Injectable	1.5 mg/m^2^ IV infusion over 24 h every 21 days; premedication with dexamethasone 20 mg IV 30 min prior; dose reductions to 1.2 or 1.0 mg/m^2^ as needed for toxicity	DNA binding; inhibition of transcription; disruption of DNA repair mechanisms; immune modulation	Headache, fatigue, asthenia, constipation, diarrhea, musculoskeletal pain, hyperpigmentation or sleep disturbances	[62,63,64]
Eribulin mesylate	Intravenous injection	1.4 mg/m^2^ administered intravenously over 2–5 min on days 1 and 8 of a 21-day cycle	Inhibition of microtubule dynamics through a mechanism distinct from other tubulin-targeting agents such as taxanes or vinca alkaloids	Nausea, constipation, anorexia, weight loss, cephalalgia, asthenia, fatigue and musculoskeletal pain, including bone, back or joint pain	[65,69]
Ziconotide	Intrathecal infusion (direct administration into the cerebrospinal fluid via an intrathecal catheter connected to an infusion pump)	2.4 µg/day (continuous intrathecal infusion)	Binding to N-type voltage-gated calcium channels	Vertigo, somnolence, nausea, cephalalgia and asthenia	[68,69]
Plitidepsin	Intravenous infusion	5 mg/m^2^ intravenously over 3 h once weekly for 3 consecutive weeks, followed by 1 week of rest (28-day cycle)	Interaction with eukaryotic translation elongation factor 1 alpha (eEF1A), involved in the elongation step of mRNA translation Inhibition of viral replication through disruption of replication complex formation and induction of eIF2α phosphorylation, inhibiting translation initiation	Fatigue, nausea and vomiting, myalgia, diarrhea, anorexia, injection-site reactions, peripheral edema and alopecia	[70,71,72]

**Table 3:** Pharmacological activities and mechanisms of action of selected bioactive compounds derived from seafood products.

Bioactive Compound	Seafood Source	Pharmacological Activity	Mechanism of Action	Evidence Basis (Model Type Reported in Cited Study)	References
Carotenoids	Shrimp, algae and seaweeds	Antioxidant and anti-inflammatory	Neutralisation of free radicals and reduction in oxidative stress and inflammation	Experimental studies including in vitro and in vivo models	[86,87]
Omega-3 Fatty Acids	Fish (salmon, tuna and sardine)	Modulation of inflammation	Regulation of inflammatory pathways through effects on cytokine production and eicosanoid synthesis	Human intervention study (supplementation-based biochemical assessment)	[88]
Polysaccharides	Green, Red and Brown Algae	Antioxidant and anticancer	Scavenging of free radicals and induction of apoptosis in cancer cells	In vitro antioxidant assays and cell-based experimental models	[89]
Saponins	Sea cucumber	Immunomodulatory and anticancer	Modulation of immune responses and induction of apoptosis in tumour cells	Cell line studies and animal experimental models	[90]
Alkaloids	Marine sponges and tunicates	Antiviral and anticancer	Inhibition of DNA topoisomerase and suppression of tumour cell proliferation following replication	Enzyme inhibition assays and in vitro cell-based studies	[91,92]
Terpenoids	Marine sponges	Antibacterial	Inhibition of bacterial growth	In vitro antibacterial screening assays	[93]
Biotoxins	Shellfish	Neurotoxic	Blockade of voltage-gated sodium channels and inhibition of action potential transmission	Molecular and toxicological studies including documented human exposure cases	[94]

**Table 4:** Cardiovascular effects of selected bioactive components derived from functional seafoods.

Bioactive Compounds	Source	Cardiovascular Effects	Pharmacological Mechanism of Action	References
Selenium	Fish and shrimp	Cardioprotective	Decrease caspase-3 and caspase-9 mRNA levels, increase Bcl-2 levels, enhance potassium channel expression and increase STAT3 activity	[101,102,103,104,105]
Omega-3 fatty acids (such as eicosapentaenoic acid)	Microalgae (*Schizochytrium*, *Crypthecodinium*), seafood	Antihypertensive, antithrombotic	Reduce blood pressure, inhibit thrombosis formation	[106]
Carotenoids (such as fucoxanthin, astaxanthin, zeaxanthin)	Algae, brown macroalgae, microalgae	Antioxidant, anti-inflammatory, anti-atherogenic	Reduce oxidative stress, inhibit inflammation, promote vasodilation and improve cardiac function	[110,112,113]
Peptides	Seafood by-products	Antihypertensive	Inhibit angiotensin-converting enzyme (ACE), induce vasodilation and decrease blood pressure	[29]
Alginate oligosaccharides	Brown seaweeds	Anti-inflammatory, antihypertensive	Reduce vascular inflammation and lower blood pressure	[113]
Vitamin D	Oily fish	Antioxidant, anti-inflammatory, antihypertensive	Reduce oxidative and inflammatory stress, improve endothelial function and regulate nitric oxide formation and the renin–angiotensin system	[109]

**Table 5:** Association of marine bioactive compounds with neurodegenerative diseases.

Compound Class	Bioactive Compound	Target Disease	Neuroprotective Effect	References
Protein	Fish protein hydrolysates	Alzheimer, Parkinson, age-related disorders	Improves learning and memory functions; Anti-inflammatory and antioxidant effect; Supporting neuronal cell viability	[119,121,122]
Polysaccharide	Fucoidan	Alzheimer, Parkinson	Reduces neuroinflammation and oxidative stress	[118,123]
	Carrageenan	Alzheimer	Protects neurons against oxidative stress; Neuroprotective and anti-inflammatory effects by inhibiting the inflammatory response of proinflammatory cytokines	[124]
	Porphyran	Alzheimer, Parkinson	Neuroprotective effect through antioxidant activity	[120,125]
Lipid	Omega-3 (DHA, EPA)	Alzheimer, Parkinson	Suppresses inflammation; Reduces amyloid beta deposition	[52]
Pigment	Astaxanthin	Alzheimer, Parkinson	Anti-inflammatory and antioxidant effects; Suppresses microglial activation and pro-inflammatory cytokines; Neuroprotective effect	[52,126,127,128]
	Fucoxanthin	Alzheimer, Parkinson	Anti-inflammatory effect; Inhibits neuroinflammation by reducing the level of inflammatory mediators	[52,127]
Polyphenols	Dieckol	Alzheimer	Anti-neuroinflammatory and antioxidant effects; Protection against neurodegeneration and neuroinflammation by inhibiting microglial activation	[52,129]

**Table 6:** Association of marine bioactive compounds with cancer.

Class of Compound	Compound Name	Types of Cancer	Anti-Cancer Effect	References
Protein	Collagen	Lung, Prostate	Antiproliferative effect; Inhibits metastasis, angiogenesis and adhesion; Reduces mRNA expression; activates apoptosis, necrosis and autophagy.	[137]
Lipid	Omega-3 fatty acids (EPA, DHA)	Breast, Colorectal, Liver, Kidney, Lung, Ovary, Pancreas, Prostate	Inhibition of tumor cell growth; Activates apoptosis; Anti-inflammatory and antioxidant effect; Inhibits angiogenesis and adhesion.	[138]
Polysaccharide	Fucoidan	Breast, Colorectal, Lung, Osteosarcoma, Ovarian, Prostate, Thyroid	Antiproliferative effect; Inhibits metastasis, angiogenesis and adhesion; decreases mRNA expression.	[139]
	Exopolysaccharide	Liver	Inhibits metastasis, invasion and adhesion	[40]
Phenolic compound	Phlorotannin	Breast, Cervix, Colorectal, Lung, Pancreas	Antiproliferative effect; Activates apoptosis; Inhibits metastasis and angiogenesis; Antioxidant effect	[19,140]
Alkaloid	Fascaplysin	Breast, Leukaemia, Lung, Prostate	Antiproliferative effect; Inhibits angiogenesis; activates apoptosis	[61,141]

## Data Availability

No new data were created or analyzed in this study. Data sharing is not applicable to this article.

## Abbreviations

The following abbreviations are used in this manuscript:

ACE	Angiotensin-converting enzyme
AD	Alzheimer’s disease
CVD	Cardiovascular diseases
DHA	Docosahexaenoic acid
EPA	Eicosapentaenoic acid
FSF	Functional seafoods
HD	Huntington’s disease
NF-κB	Nuclear factor kappa B
Nrf2	Nuclear factor erythroid 2–related factor 2
PD	Parkinson’s disease
ROS	Reactive oxygen species
